# Efficacy of High-Volume Low-Concentration Intraperitoneal Bupivacaine Irrigation for Postoperative Analgesia in Patients Undergoing Laparoscopic Cholecystectomy: Bupivacaine Irrigation for Analgesia

**DOI:** 10.1155/2024/4545400

**Published:** 2024-10-30

**Authors:** Swati Panwar, Mona Arya, J. S. Dali, Kapil Chaudhary, Sushanto Neogi

**Affiliations:** ^1^Department of Anaesthesiology and Intensive Care, Maulana Azad Medical College and Associated Lok Nayak Hospital, New Delhi, India; ^2^Department of Surgery, Maulana Azad Medical College, New Delhi, India

**Keywords:** analgesia, bupivacaine, cholecystectomy, laparoscopic, peritoneal cavity

## Abstract

**Background:** Intraperitoneal irrigation with a low-volume, high-concentration local anaesthetic in laparoscopic cholecystectomy (LC) provides less pain relief. We investigated the impact of high-volume, low-concentration bupivacaine on postoperative pain and opioid requirement.

**Methods:** Patients undergoing LC were randomised into Group B (20 mL of 0.5% bupivacaine in 480 mL normal saline) or Group S (500 mL of normal saline). Fifteen patients were included in both the groups but one patient was excluded from Group S because of bile duct injury. The primary outcome was Duration of Analgesia (DOA). The secondary outcomes were the Numeric Pain Rating Scale (NRS) at extubation, at 15 min, 30 min and 1, 2, 4, 8, 12 and 24 h. Cumulative rescue analgesics, incidence of postoperative nausea, vomiting and shoulder pain.

**Results:** Mean (median/range) duration of analgesia was 6.45 ± 5.57 h (6/0.15–24) in Group B vs 3.18 ± 4.21 h (0.3/0.15–12) in Group S. Cumulative requirement of rescue analgesic was higher in saline group being 56.25 ± 33.92 mg for diclofenac and 83.57 ± 66.75 mg for tramadol vis-à-vis 40.9 ± 39.17 mg and 30.00 ± 52.78 mg, respectively, in bupivacaine group.

**Conclusion:** Irrigation of the peritoneal cavity with high-volume low-concentration bupivacaine in LC increases the duration of analgesia and decreases the analgesic requirement in the postoperative period.

**Trial Registration:** ClinicalTrials.gov identifier: CTRI/2019/02/017802 dated 25/02/2019.

## 1. Introduction

Laparoscopic cholecystectomy (LC) is associated with less postoperative pain than conventional open cholecystectomy [1]. Still, pain remains the predominant complaint in the initial 24 h postoperative period [2, 3]. Most of the studies have used low-volume (20 mL to 100 mL) high-concentration (0.125%–0.5%) bupivacaine for irrigation of peritoneal cavity. However, the analgesic action of these Local Anaesthetics (LAs) lasts for a few hours only [4–7].

Recently [8], low concentration (0.02%) and high volume (500 mL) of bupivacaine have been used for intraperitoneal irrigation during LC to cover a larger subhepatic area and surrounding peritoneum thereby producing more effective analgesia. Intraperitoneal irrigation with high-volume low-concentration bupivacaine significantly increased the duration of analgesia and reduced opioid requirement after LC.

Thus, we hypothesized that intraperitoneal irrigation with high-volume (500 mL) low-concentration (0.02%) bupivacaine is more efficacious for postoperative analgesia in patients undergoing LC.

## 2. Methods

### 2.1. Study Design and Ethics

After institutional review board approval and informed written consent from the patient, a double-blind randomised controlled study was conducted over a period of 1 year (January to December 2021). The clinical research was done in accordance with the ethical principles for medical research involving human subjects, outlined in the Helsinki Declaration of 1975 (revised 2013). Randomization was performed using a computer-generated random number technique and patients were divided into two groups of 15 patients each, that is, Group S and Group B. In Group B 20 mL of 0.5% (100 mg) bupivacaine in 480 mL of normal saline (total volume 500 mL) was used for intraperitoneal irrigation whereas in Group S, 500 mL of normal saline was used as irrigating fluid. According to randomization, the sealed envelope was opened by the anaesthesiologist, who was not involved in the study and then prepared the drug solution in identical bottles in both groups.

### 2.2. Participants

American Society of Anaesthesiologist (ASA) grade I/II patients of either sex, between 20 and 60 years of age and scheduled for elective LC under general anaesthesia were included whereas patients allergic to local anaesthetics, having acute pancreatitis, choledocholithiasis, current analgesic use, inability to comprehend Numeric Pain Rating Scale 0–10 (NRS) and pregnant women were excluded.

### 2.3. Anaesthesia and Perioperative Care

A detailed preanaesthetic evaluation including history, examination (including airway assessment) and investigations as required for age and diagnosis were performed. The demographic profile including age, sex, weight, height and body mass index (BMI) was recorded. The anaesthesia technique was standardised for all patients. All patients fasted for 6 h before surgery. On arrival in the operating theatre, standard monitors (ECG, pulse oximeter and noninvasive blood pressure) were attached and baseline values were recorded. All patients were premedicated with intravenous injection of Midazolam 0.025 mg/kg and Fentanyl (2 *μ*g/kg). General anaesthesia was induced with injection of Propofol 2 mg/kg IV and injection of Vecuronium bromide 0.1 mg/kg IV was used to facilitate the insertion of the airway device according to the discretion of the anaesthesiologist. Anaesthesia was maintained with Isoflurane 0.8%–1% in a mixture of 33% oxygen and nitrous oxide. Intraoperatively, mean arterial pressure (MAP) and heart rate (HR) were recorded every 5 min. Intra-abdominal pressure was restricted to ≤ 12 mmHg throughout the surgery.

The surgeon used the irrigation fluid as per group allocation. The remaining irrigating fluid was used to irrigate the surgical bed and peritoneal cavity after the gall bladder extraction. The patient was placed in the right Trendelenburg position for 5 min to facilitate the dispersion of the drug solution in the subhepatic region. The irrigating fluid was aspirated through the subcostal trocar under direct laparoscopic control and the surgical ports were closed.

A standardised anaesthesia technique was used for the reversal (Injection Neostigmine 50 *μ*g/kg and Injection Glycopyrrolate 10 *μ*g/kg) of anaesthesia. Patients for whom the irrigation fluid requirement exceeded 500 mL and LC was converted to open cholecystectomy were excluded from the study.

For intraoperative analgesia, if required, injection Fentanyl 0.5 mg/kg IV supplementation was administered intravenously but not within 30 min of surgery. All patients also received injection Diclofenac sodium 1.5 mg/kg IV (maximum dose never exceeded 150 mg) and injection Ondansetron 0.1 mg/kg IV 30 min before the expected completion of surgery. No further analgesics were administered during surgery.

The following parameters were recorded postoperatively.

#### 2.3.1. Primary Outcome

i. Duration of Analgesia, defined as the time interval between removal of the airway device and requirement of the first rescue analgesic.

#### 2.3.2. Secondary Outcome

i. NRS at fixed intervals, that is, immediately after extubation, and at 15 min, 30 min, 1 h, 2 h, 4 h, 8 h, 12 h and 24 h. Injection diclofenac 1.5 mg/kg IV was administered as a rescue analgesic whenever NRS > 3 or if demanded by the patient. If the pain was not relieved within 30 min of first rescue analgesic, second rescue analgesic, i.e., injection tramadol 2 mg/kg IV was administered. Injection diclofenac was not given within 6 h of the last dose which every patient received half an hour before completion of surgery and the total dose of diclofenac never exceeded 150 mg in 24 h. If the patient was found to be sleeping, he or she was presumed to be pain free (NRS = 0) and not disturbed for assessment.
ii. Cumulative requirement of rescue analgesic in 24 h postoperative period.
iii. Incidence of shoulder pain, nausea and vomiting.

### 2.4. Statistical Analysis

The sample size was calculated based on the findings of a previous study by Jain et al. [8] which reported analgesia of 19.35 ± 8.64 h in the bupivacaine (study) group in comparison to 0.06 ± 0.172 h in the normal saline (control) group. Assuming the given durations as reference values, the minimum required sample size at a 5% level of significance and 95% power was 3 patients per group. However, we included 15 patients in each group to improve the power of the study.

(1) $$\begin{array}{c} n=\frac{2\sigma^{2}{\left(z_{\alpha /2}+z_{\beta }\right)}^{2}}{{∆}^{2}}, \end{array}$$

where *n* is the number of subject required in each group, Δ is the expected difference and *z* is cumulative normal distribution at *α*/2 level of significance, *z* is cumulative normal distribution at *β* value (where power = 1 − *β*), and *σ* is the standard deviation of the difference.

**Figure 1:**
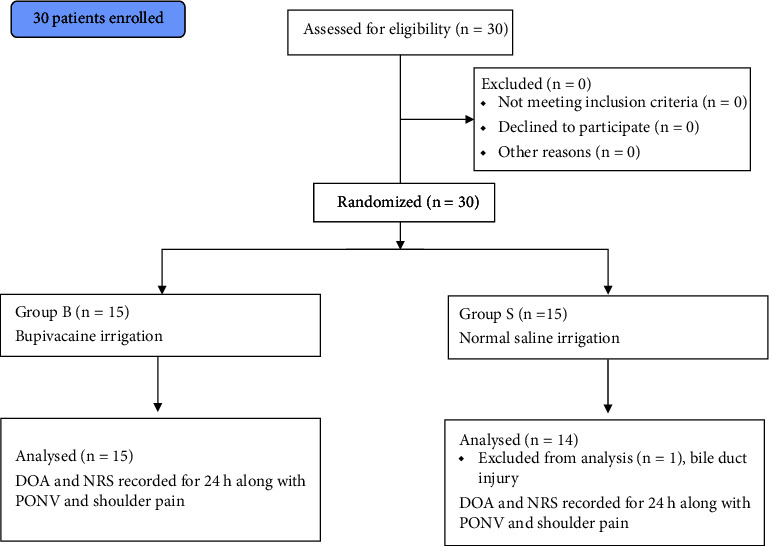
CONSORT 2010 flow diagram.

The collected data was thoroughly screened, evaluated in MS Excel spreadsheet and statistical analysis was performed using “*R*”-programming language. Quantitative variables were compared between groups using the unpaired and paired *t*-tests. Qualitative variables were compared using the Fisher's exact test. Statistical significance was set at *p* < 0.05. A CONSORT flow diagram has been included providing information on conduction, enrolment, allocation, follow-up and analysis of patients involved in this study. (see Figure 1).

## 3. Results

**Figure 2:**
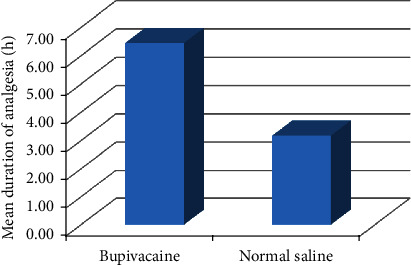
Mean duration of analgesia in two groups.

The analysis did not reveal any significant difference between two groups in terms of demography. All the studied vitals were statistically insignificant too as shown in Table 1. One patient was excluded from Group S while comparing the data because of unbearable pain during the postoperative period. On follow-up, we found that patient had common bile duct injury and endoscopic clipping was performed for the same. The mean (median/range) duration of analgesia was found to be 6.45 ± 5.57 h (6/0.15–24) in Group B whereas 3.18 ± 4.21 h (0.3/0.15–12) in Group S (*p* 0.044) as highlighted in Table 1 and Figure 2.

**Figure 3:**
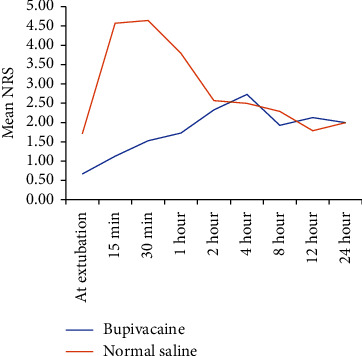
Mean NRS in two groups.

At extubation, the mean/median NRS was higher in Group S as seen in Figure 3. In Group S, 6 patients required rescue analgesic immediately after extubation and 10 patients within 30 min of extubation whereas in Group B only one patient required rescue analgesia immediately after extubation. In Group S, nine patients required a second dose of rescue analgesic, while only two patients in Group B required a second dose of rescue analgesic over 24 h. The mean NRS value became equivalent at around 4 h after extubation. In comparison, the *p* value (0.006) was significant only till 1 h post extubation as shown in Table 2.

**Figure 4:**
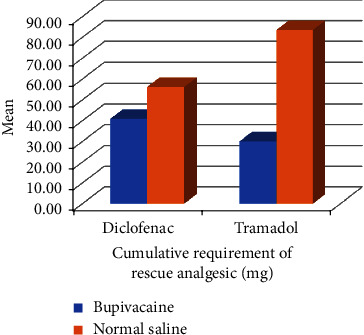
Mean cumulative requirement of rescue analgesic.

**Figure 5:**
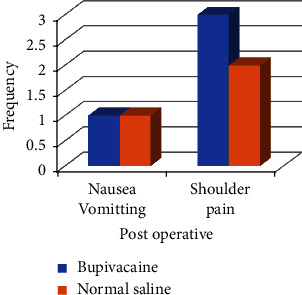
Incidence of postoperative nausea vomiting and shoulder pain.

There were no significant differences in terms of the total mean dose of diclofenac in either group (*p* 0.163). However, the difference was significant (*p* 0.012) in case of mean requirement of tramadol. Over all mean cumulative requirement of rescue analgesic was higher in saline group as compared to that of bupivacaine group in postoperative period as shown in Figure 4. This came out to be 56.25 ± 33.92 mg in Group S for diclofenac and 83.57 ± 66.75 mg for tramadol as compared to that of 40.9 ± 39.17 mg and 30.00 ± 52.78 mg, respectively, in Group B as can be seen in Table 1. A single patient in both groups had PONV whereas 3 and 2 patients reported shoulder pain in Groups B and S, respectively, as highlighted in Figure 5. Table 1 shows that *p* value was 0.480 for PONV and 0.342 for shoulder pain between the two groups.

## 4. Discussion

We found that peritoneal irrigation with high volume low concentration bupivacaine provides analgesia for up to 6 h in postoperative period with less requirement of rescue analgesic along with lower incidence of PONV and shoulder pain.

LC has achieved outcomes superior to those of conventional open cholecystectomy in terms of reduced postoperative pain, short hospital stay, early return to regular activity and cosmesis. Pain in open cholecystectomy is parietal whereas in LC, it is multifactorial [2, 3], requiring multimodal analgesia. Three types of pain have been reported following LC, i.e., parietal pain caused by the trocar incision; visceral pain [6], caused by dissection of gall bladder, tearing of blood vessels and traction on the nerves and referred pain at the shoulder tip due to diaphragmatic irritation caused by residual CO_2_. Visceral pain is a major contributor to pain.

Many studies [9–12] have been performed utilizing low-volume, high-concentration LA for intraperitoneal instillation in LC but postoperative pain still remains a challenge.

Jain et al. [8] used high-volume, low-concentration intraperitoneal bupivacaine for post-LC analgesia in 60 patients after randomization into two groups. Postoperative pain, duration of analgesia and opioid requirement were evaluated and they concluded significantly increased postoperative duration of analgesia and reduced opioid requirement.

Patients already being on analgesics for their underlying illness would have influenced proper pain assessment during the postoperative period, thus, excluded. Various studies [13–15] have proved an association between persistent analgesic use and an increase in baseline pain sensitivity along with reduced pain tolerance for both opioid and nonopioid analgesics.

Although surgeons experience greater difficulty in dissection during low-pressure pneumoperitoneum, it is better in terms of postoperative pain and analgesic requirements. Pain is worse when high pressure is involved and sympathetic hyperactivity is seen as well. Beqiri et al. [16] compared high pressure (15 mmHg) with low pressure (10 mmHg) pneumoperitoneum with or without bupivacaine infiltration. They concluded that combination of low pressure and local infiltration reduced pain intensity and opioid consumption after LC. Therefore, to eliminate confounding factors we restricted the intra-abdominal pressure to 12 mmHg.

Same surgical team was included for every case in order to avoid discrepancies in the result. The rate of topical wash with LA over the liver and gall bladder was higher in emergency laparoscopic cholecystectomies and thus did not give enough time to LA to initiate its action. A plausible explanation given by Robert et al. [9] for insignificant pain score was the longer duration of surgery and inflammation associated with cholecystitis and pancreatitis.

The DOA postoperatively was longer in the bupivacaine group in comparison to the saline group in our study (*p* = 0.044). In the study conducted by Jain et al. [8], the duration of analgesia was 19.35 ± 8.64 h in the bupivacaine group and 0.06 ± 0.172 h in the saline group. By virtue of the study protocol of Jain et al. [8] wherein all patients received injection diclofenac 1.5 mg/kg iv at 8 h and 16 h routinely in postoperative period and only cumulative requirement of injection tramadol was observed which patients received on demand in case of inadequate analgesia. A feasible justification for a much longer duration of analgesia in bupivacaine group is that all patients received a predetermined dose of diclofenac at 6 h after extubation, thus cloaking the actual analgesic duration assessment provided by intraperitoneal irrigation with high-volume low-concentration bupivacaine. If we had administered diclofenac 8 hourly, then the duration of analgesia would have been much longer.

Our result were consistent with those of Alam et al. [10] and Castillo-Garza et al. [7] in providing pain free environment for up to 6 h.

Cunniffe et al. [11] used high volume (10 mL of 0.5% in 500 mL saline) bupivacaine to irrigate bilateral subdiaphragmatic area at the end of surgery for postoperative analgesia and provided evidence in favour of our study. Mraovic et al. [12] found that intraperitoneal bupivacaine reduced postoperative pain after LC up to 8 h. They put forward the evidence of dividing the dose of LA into two, the first half to be administered before surgical intervention so as to block shoulder pain and the second dose to reduce visceral pain after gall bladder removal. They concluded that low volume low-concentration bupivacaine loses its potential analgesic action due to its distribution in large peritoneal cavity [17]. Elfberg and Sjövall-Mjöberg [18] used 2 mg/kg bupivacaine for intraperitoneal instillation at the end of surgery and concluded that there was no decrease in analgesic requirement probably because the dose of bupivacaine was too small.

Our conclusion was furthered by Nunez et al. [19], who compared small volume high concentration with large volume low concentration levobupivacaine in brachial plexus block. The complete sensory block was higher in large volume group than high concentration group. Local anaesthetics deposited in subdiaphragmatic area tends to move away from the cholecystectomy wound because of anatomic intraperitoneal flux [20]. In order to overcome this flaw Verma et al. [21] placed surgical soaked with bupivacaine in gall bladder fossa thus proving its effectiveness in the management of postoperative pain after surgery.

Gupta and Hopkins [22] demonstrated that it is the mass and not the concentration of bupivacaine which determines ED_50_ for achieving supraclavicular brachial plexus block. The success rate was lower in patients receiving low volume high concentration than in those receiving high volume low concentration. The above-mentioned studies furthered our study, thus, favouring the use of high volume low concentration LA for irrigation of peritoneal cavity for better pain relief in LC.

NRS scores were significant up to 1 h (*p* = 0.010) and at 8 h (*p* = 0.051) after extubation. The mean pain score at extubation was higher in the saline group (1.71 ± 1.2) than in the bupivacaine group (0.67 ± 0.9). The median NRS among two groups was significant till 1 h postextubation with *p* value < 0.05 at each time interval. In the study by Jain et al. [8], the NRS score at extubation was 4.67 ± 1.2 in Group S while in Group B it was 0.47 ± 0.77 (*p* 0.000), reason being, all patients in Group S required rescue analgesic within half-hour of extubation. The NRS score was insignificant at other time intervals due to administration of rescue analgesic in immediate postoperative period which brought down NRS scores in Group S at subsequent time intervals [8]. Chundrigar et al. [23] demonstrated that intraperitoneal administration of 20 mL of 0.25% bupivacaine was effective in reducing pain in early postoperative period (1 h and 2 h). Pain scores were lower in both groups at 4 h and at subsequent time intervals.

Overall mean cumulative requirement of rescue analgesic was higher in Group S. Our finding coincides well with the finding of Jain et al. [8] in which the cumulative requirement of tramadol in 24 h in Group S was 123.33 ± 43.01 mg which was significantly higher (*p* 0.00) than that in Group B (23.33 ± 43.01 mg). Most of the patients in Group S required analgesics in immediate postoperative period which decreased the pain scores at subsequent time intervals. In addition, three patients required a single dose of diclofenac in 24 h period and 2 patients required two doses in Group S in our study. Three patients required only a single dose of tramadol in 24 h period. Six of 14 patients received both diclofenac and tramadol within 24 h in Group S. In our study, six of 15 patients received only a single dose of diclofenac in Group B. Four patients required tramadol in postoperative period and four patients received two doses of diclofenac. One patient received neither of the rescue analgesics and had a DOA of 24 h postextubation. In a study by Jain et al. [8], 26 of 30 patients required rescue analgesics immediately after extubation and the remaining four patients within half an hour postoperatively in Group S and three patients required a second dose of rescue analgesic in 24 h period. In Group B, only one patient required rescue analgesics within half an hour postoperatively. Overall, only seven of the 30 patients required rescue analgesic in 24 h and no patient required any additional dose.

Zmora et al. [24] observed no significant difference in the average pain levels although the average analgesic requirement was lower in the bupivacaine group (100 mg in 50 cc saline) but did not reach statistical significance. Thus, showing results similar to those of present study.

In our study, the overall incidence of shoulder pain was 16.66% in both the groups but not statistically significant (*p* 0.342). This correlates with Jain et al. [8], where six patients in both the groups had shoulder pain with incidence of 20% (*p* 0.738). Pneumoperitoneum induces peritoneal hypoxia which leads to metabolic acidosis by an immediate drop to pH 6.6 and stabilizes at 6.4. Gas insufflation causes abdominal distension and stretching of subdiaphragmatic fibres due to increased concavity of diaphragm and phrenic nerve neuropraxia of short duration [25]. In a study conducted by Cunniffe et al. [11], the incidence of shoulder tip pain was found to be of 42% in control group vis-à-vis 7% in study group, reducing postoperative analgesic requirement. They suggested that intraperitoneal irrigation with bupivacaine to both hemidiaphragms at the end of surgery significantly reduced both frequency and intensity of shoulder tip pain following laparoscopic procedures.

We found lower PONV incidence (6.66%) in both the groups. A possible explanation could be the use of antiemetic before completion of surgery. The incidence of PONV is higher after laparoscopic surgeries but it has no correlation with irrigation of peritoneal cavity with local anaesthetic. Jain et al. [8] demonstrated the incidence of PONV to be 16.66%. Five patients in each group developed PONV (*p* 0.905).

The findings are consistent with previous studies concluding that the cumulative requirement of rescue analgesics is lower with intraperitoneal instillation of bupivacaine than with saline. However, this did not reach statistical significance because in the saline group, patients required rescue analgesics in the immediate postoperative period. However, the requirement of this dose gets is delayed by 4 h to 6 h in bupivacaine group which may vary with patient. Subsequent doses of rescue analgesics depended on patient's pain scores within 24 h. This further depends upon clear dissection of the gall bladder, whether the surgeon has encountered a clear surgical field or a field filled with adhesions or fibrosis. This might be observed in patients with history of any previous surgery. Any event of bile leak during the intraoperative period can result in postoperative pain. Although we tried to control this factor by pertaining to one surgical team and being a teaching institution various residents performed the surgery, thus, this was beyond our control.

### 4.1. Limitations

We did not study the analgesic effect of local anaesthetic on dynamic pain only static pain was covered. We did not take low volume high concentration into consideration keeping blinding into account. We studied generalised abdominal pain rather than parietal or visceral pain. Only the incidence of shoulder pain was observed and not scored.

However, to validity the discernibility of our findings, further studies are required to evaluate the contribution of high-volume low-concentration bupivacaine for intraperitoneal irrigation in LC to attain analgesia postoperatively. Further, amelioration of our study can be done by giving pre-emptive local anaesthetic infiltration at trocar site and dynamically assessing all three sites of pain; that is, parietal, visceral and shoulder tip pain.

We conclude that irrigation of peritoneal cavity with high volume and low concentration of bupivacaine during LC increases the duration of analgesia and decreases the analgesic requirement in the postoperative period. Additionally, it is associated with a lower incidence of postoperative nausea and shoulder pain.

## Figures and Tables

**Table 1:** Demography, haemodynamic and outcome.

Gender	Bupivacaine (*n* = 15)		Normal saline (*n* = 14)		*p* value
	*n*	%	*n*	%	
Male/female	1/14		4/10		0.059
ASA status					
I	13	86.67	9	64.29	0.080
II	2	13.33	5	35.71	
Total	15	100	14	93	
Device used for intubation					
ETT	2	13.33	1	7.14	0.292
PLMA	12	80.00	11	78.57	0.462
I-gel	1	6.67	0	0.00	0.163
Supreme LMA	0	0.00	1	7.14	0.146
Baska	0	0.00	1	7.14	0.146
Postoperative					
Nausea vomiting	1	6.67	1	7.14	0.480
Shoulder pain	3	20.00	2	14.29	0.342

	**Bupivacaine**		**Normal saline**		
	**Mean**	**±sd**	**Mean**	**±sd**	

Duration of surgery (mins)	116.00	±25.01	112.00	±22.6	0.348
Pulse Rate (bpm)	79.80	±10.09	80.86	±14	0.408
Systolic BP (mm Hg)	124.67	±6.91	126.86	±10.88	0.363
Diastolic BP (mm Hg)	80.40	±4.97	80.64	±8.02	0.461
SpO_2_ (%)	100.00		100.00		
Duration of analgesia (h)	6.45	±5.57	3.18	±4.21	0.044
Cumulative requirement of rescue analgesic (mg)					
Diclofenac	40.91	±39.17	56.25	±33.92	0.163
Tramadol	30.00	±52.78	83.57	±66.75	0.012

*Note:* Bupivacaine group = 20 mL of 0.5% bupivacaine with 480 mL normal saline for intraperitoneal irrigation. Normal saline group = 500 mL of normal saline for intraperitoneal irrigation. Statistical tests used—paired and unpaired *t* test. % = percentage.

Abbreviations: bpm = beats per minute, ETT = endotracheal tube, h = hour, LMA = laryngeal mask airway, mins = minutes, mmHg = millimetres of mercury, mg = milligram, *n* = number of patients, sd = standard deviation.

**Table 2:** Mean NRS in two groups.

NRS	Bupivacaine			Normal saline			*p* value
	Mean ± sd	Median (IQR)	*p* value vs extubation	Mean ± sd	Median (IQR)	*p* value vs extubation	
At extubation	0.67 ± 0.9	0 (1)	—	1.71 ± 1.2	2 (2)	—	0.006
15-min	1.13 ± 2.1	0 (1)	0.093	4.57 ± 4.01	3.5 (7.75)	0.002	0.003
30-min	1.53 ± 2.07	1 (1)	0.021	4.50 ± 3.57	6 (5.5)	0.001	0.005
1-h	1.73 ± 1.03	2 (1)	< 0.001	3.07 ± 1.82	3 (2)	0.003	0.010
2-h	2.33 ± 0.62	2 (1)	< 0.001	2.43 ± 0.85	2 (1)	0.018	0.366
4-h	2.73 ± 0.88	3 (1)	< 0.001	2.29 ± 0.73	2 (2)	0.089	0.075
8-h	1.93 ± 1.1	2 (1)	< 0.001	2.79 ± 1.58	2.5 (3)	0.039	0.051
12-h	2.13 ± 1.3	2 (1)	0.003	1.93 ± 1.21	2 (1)	0.320	0.332
24-h	2.00 ± 0.85	2 (2)	< 0.001	2.00 ± 0.68	2 (0)	0.195	0.500

Abbreviation: IQR = inter quartile range.

## Data Availability

Data supporting the results of our study are available with the primary author.
